# *trans-*11 18:1 Vaccenic Acid (TVA) Has a Direct Anti-Carcinogenic Effect on MCF-7 Human Mammary Adenocarcinoma Cells

**DOI:** 10.3390/nu6020627

**Published:** 2014-02-10

**Authors:** Ji-Na Lim, Jin-Ju Oh, Tao Wang, Jae-Sung Lee, Sang-Hun Kim, Yoon-Jin Kim, Hong-Gu Lee

**Affiliations:** 1 Department of Animal Science and Technology, College of Animal Bioscience & Technology, Konkuk University, Seoul 143-701, Korea; E-Mails: ooopxoopx@naver.com (J.-N.L.); foodleeking@gmail.com (J.-S.L.); 2 Natural Product Clinical Research Center, Clinical Research Center, Pusan National University School of Medicine, Busan 602-739, Korea; E-Mail: chcasa7@nate.com; 3 College of Animal Science and Technology, Jilin Agricultural University, Changchun 130118, China; E-Mail: cagewang@163.com; 4 Key Laboratory of Animal Nutrition and Feed Science, Jilin Agricultural University, Changchun 130118, China; 5 Department of Biology, Kyung Hee University, Seoul 130-701, South Korea; E-Mails: shkim@khu.ac.kr (S.-H.K.); shkim@khu.ac.kr (Y.-J.K.)

**Keywords:** apoptotic protein, cancer cells, *cis*-9, *trans*-11conjugated linoleic acid, stearoyl CoA desaturase, trans fatty acids

## Abstract

Trans vaccenic acid (TVA; *trans*-11 18:1) is a positional and geometric isomer of oleic acid and it is the predominant trans isomer found in ruminant fats. TVA can be converted into *cis*-9, *trans*-11 conjugated linoleic acid (c9, t11-CLA), a CLA isomer that has many beneficial effects, by stearoyl CoA desaturase 1 (SCD1) in the mammary gland. The health benefits associated with CLA are well documented, but it is unclear whether trans fatty acids (TFAs) from ruminant products have healthy effects. Therefore, the effects of TVA on the proliferation of MCF-7 human breast adenocarcinoma cells and MCF-10A human breast epithelial cells were investigated in the present study. Results showed that TVA inhibited the proliferation of MCF-7 cells but not MCF-10A cells by down-regulating the expression of Bcl-2 as well as procaspase-9. In addition, the suppressive effect of TVA was confirmed in SCD1-depleted MCF-7 cells. Our results suggested that TVA exerts a direct anti-carcinogenic effect on MCF-7 cells. These findings provided a better understanding of the research on the anti-carcinogenic effects of TVA and this may facilitate the manufacture of TVA/c9, t11-CLA fortified ruminant products.

## 1. Introduction

Trans fat is the common name for unsaturated fat containing *trans*-isomer fatty acids. The health risks associated with consumption of diets high in trans fats from commercially hydrogenated fats are well documented [1]. However, little is known about the health effects of ruminant trans fats [2]. Trans vaccenic acid (TVA, *trans*-11 18:1), a positional and geometric isomer of oleic acid which is predominantly found in ruminant-derived products. This isomer comprises about 60%-80% of the total natural ruminant trans fats [3]. TVA is the dietary precursor of *cis*-9, *trans*-11 conjugated linoleic acid (c9, t11-CLA). Recent study has suggested TVA has a beneficial effect in reducing the incidence of heart disease, cancer, and obesity [3].

CLA is predominantly found in food from ruminant products such as milk, beef, and lamb. This compound has a lot of positive activities including anti-diabetic, fat reducing, and anti-carcinogenic effects [3,4,5,6]. It was found that a dose-dependent increase in tissue concentrations of c9, t11-CLA occurs in response to TVA consumption, and this is accompanied by a progressive reduction in premalignant lesions in mouse’s mammary glands [7]. It has also been suggested that TVA has health benefits beyond those related with CLA [3,8]. *In vitro* study has shown that treatment with vaccenic acid inhibits the growth of HT-29 human colonic adenocarcinoma cells [2]. Increased DNA fragmentation and reduced cytosolic glutathione levels were observed in MCF-7 and SW480 following 4 days of treatment with pure vaccenic acid (20 μg/mL) [9]. Molecular mechanisms underlying the effects of TVA and CLA on cancer cells are still not clear. Several studies have suggested that CLA induces apoptosis by down-regulating the expression of apoptotic factors [10,11,12,13]. CLA could induce time- and concentration-dependent cleavage of caspases-3 and -9, and cytochrome c release from mitochondria into the cytosol [14].

The anti-carcinogenesis effect of TVA is direct or via the conversion to c9, t11-CLA is still unclear. Therefore, in this study the effects of TVA on the proliferation of MCF-7 human breast adenocarcinoma cells and MCF-10A human breast epithelial cells were investigated.

## 2. Experimental Section

### 2.1. Cell Culture

MCF-7 cells (ATCC HTB 22) that had been isolated from a female patient with breast adenocarcinoma were obtained from the American Type Culture Collection (Manassas, VA, USA) [15]. This human cancer cell line was chosen because of the high impact of cancer on the human mortality [16,17,18]. The cells were maintained in Dulbecco’s modified Eagle medium (DMEM, Thermo Fisher Scientific Inc., Waltham, MA, USA) supplemented with 10% heat-inactivated fetal bovine serum (FBS, Thermo Fisher Scientific Inc.), 5 μg/mL bovine insulin (Sigma-Aldrich, St. Louis, MO, USA), and 1% penicillin-streptomycin (Thermo Fisher Scientific Inc.). MCF-10A cells were also obtained from the ATCC (CRL 10317). The cell line was derived from normal human mammary gland [19]. The cells were maintained in Dulbecco’s Modified Eagle Medium:Nutrient Mixture F-12 (DMEM/F-12, Invitrogen Co., Carlsbad, CA, USA) supplemented with 5% fetal horse serum (Thermo Fisher Scientific Inc.), 1% penicillin-streptomycin (Thermo Fisher Scientific Inc.), 10 μg/mL bovine insulin (Sigma-Aldrich), 20 ng/mL Epidermal growth factor (EGF, Sigma-Aldrich), 0.5 mg/mL hydrocortisone (Sigma-Aldrich), and 100 ng/mL cholera toxin (Sigma-Aldrich). All the cells were incubated at 37 °C in a humidified atmosphere 95% air and 5% CO_2_.

### 2.2. Cell Proliferation Assay

Cell proliferation was evaluated using an MTT assay. In general, the cells were seeded at a density of 5 × 10^3^ cells/well in 96-well plates (Corning Inc., Lowell, MA, USA) and incubated (37 °C with 5% CO_2_) overnight to allow the cells to attach to the wells. After that cells treated with fatty acid-free bovine serum albumin (0.5 mM BSA, vehicle; Sigma-Aldrich), TVA (50–200 μM; ≥99%, capillary GC; Sigma-Aldrich), and c9, t11-CLA (50–200 μM; ≥98%, GC; Matreya LLC, Pleasant Gap, PA, USA) for 72 h. TVA and c9, t11-CLA were dissolved in 0.5 mM BSA prior to the treatment. Thiazolyl blue tetrazolium bromide (5 mg/mL; Sigma-Aldrich) dissolved in PBS was added into each well, and the cells were incubated at 37 °C for 4 h. The medium was removed, and 200 μL of DMSO (Sigma-Aldrich) was added to each well. The plate was gently rotated on an orbital shaker for 15 min to completely dissolve the precipitants. Absorbance was measured at 550 nm with a Multiskan EX Microplate Reader (Thermo Fisher Scientific Inc.).

### 2.3. Protein Sample Preparation

Total cell proteins were obtained from cells grown in 100-mm dishes for 72 h after being treated [20,21]. Briefly, the cells were washed three times with ice-cold PBS after which they were lysed on ice in lysis buffer containing 8 M urea, 4% CHAPS, 5 mM EDTA, 10 mM Tris-HCl (pH 8.3), and protease inhibitors (GE Healthcare Corp., Piscataway, NJ, USA). The lysates were then transferred to a plastic centrifuge tube and sonicated on ice with a probe-tip sonicator using 2 s bursts with 5 min pauses between sonication cycles to prevent overheating. The supernatant was then collected after centrifugation at 16,600× *g* at 20 °C for 15 min. Protein concentration was then determined using a PlusOne 2-D Quant Kit (GE Healthcare Corp.) after which aliquots of the samples were stored at −80 °C until needed.

### 2.4. Expression of Pro-Apoptotic Factors

The expression of proteins involved in apoptosis was analyzed by Western blotting. The cells was washed twice with PBS and detached with trypsin (0.25% for 10 min, Thermo Fisher Scientific Inc.). The cells were then scraped into cold Radio Immuno-precipitation Assay (RIPA) buffer (pH7.4, 10 mM Tris-HCl (pH 7.5), 1 mM EDTA, 150 mM NaCl, 1% Nonidet P-40, 2% SDS, 1% sodium deoxycholate, 50 mM NaF, 0.2 mM Na_3_VO_4_, 1 mM PMSF, 1% protease inhibitor mixture) and centrifuged at 2000× *g*, 4 °C for 20 min. The supernatants were recovered and the proteins (15 μg protein/lane) were separated by 10% SDS-PAGE. Proteins were transferred to a polyvinylidenedifluoride membrane (GE Healthcare Corp.) and the membrane was then blocked with 5% skim milk dissolved in PBS-T (BD Biosciences, San Jose, CA, USA) for 1 h at room temperature. The membrane was incubated overnight with the first antibodies against β-actin (1:1000; Abcam, Inc., Cambridge, MA, USA), Bcl-2 (1:175; Santa Cruz Biochemistry, Santa Cruz, CA, USA), procaspase-8 (1:500; BD Biosciences), or procaspase-9 (1:450; Calbiochem, San Diego, CA, USA) at 4 °C. After three washing with TBS-T, the membrane was incubated with a horseradish peroxidase (HRP)-conjugated anti-mouse IgG (1:1000; Abcam Inc., Cambridge, MA, USA) for 30 min at room temperature. The membrane was then washed and antibody binding was visualized with an enhanced chemiluminescence (ECL) system plus detection kit (GE Healthcare Corp.). The membrane was then exposed to X-ray film (Fujifilm Corporation, Minato-ku, Tokyo, Japan) for 1–3 min. The bands were quantified using the Image J 1.43 software (Image J, NIH image, Bethesda, MD, USA).

### 2.5. SCD Protein Knockdown

Specific small interfering RNA (siRNA) was synthesized by Bioneer Corporation (Daejeon, South Korea) and was transfected into cells using the Lipofectamine^®^ RNAIMAZ Reagent (Invitrogen Co.) according to the manufacturer’s recommendations. Briefly, the cells were grown in medium (DMEM, Thermo Fisher Scientific Inc.) without antibiotics one day before the experiment. On the day of transfection, pre-prepared oligonucleotide solutions was mixed with Oligofectamine reagents dissolved in medium without serum or antibiotics, and incubated for 15–20 min at room temperature. Meanwhile, the medium of the cells prepared for SCD knockdown was removed, and replaced with medium without serum or antibiotics. Then the cells were incubated with the oligonucleotide/oligofectamine solution at 37 °C for 48 h. The following siRNA molecules were used in this study: CCTACGCCACCAATTTCGT (scrambled) as the control, GAGAUAAGUUGGAGACGAU (siRNA1) and GAUAUGCUGUGGUGCUUAA (siRNA2), GGGCATAACAGCAGGAGCT (siRNA3), and GGAGATGGAAACTACAAGA (siRNA4). In addition, the cell proliferation after siRNA transfection was evaluated by MTT assay as described above.

### 2.6. mRNA Isolation and Quantitative Real-Time PCR

Total RNA was extracted from cells using Trizol reagent (Invitrogen Co.) according to the manufacturer’s instructions after 48 h of siRNA transfection. The RNA (3 μg) was then used as a template for single-stranded cDNA synthesis, which was conducted by incubating the RNA and Moloney Murine Leukemia Virus Reverse Transcriptase (MMLV reverse transcriptase, Invitrogen Co.) at 37 °C for 1 h. The primers specific for stearoyl-CoA desaturase-1 (SCD1) and β-actin (as the housekeeping gene) were 5′-GGATGCAGAAGGAGATCACTG-3′ (sense) and 5′-CGATCCACACGGAGTACTTG-3′ (antisense) for SCD1, 5′-GAAGGGGAGTACGCTAGACTTGT-3′ (sense) and 5′-ACATCATCAGCAAGCCAGGT-3′ (antisense) for β-actin. The following PCR program was used: initial denaturation at 95 °C for 3 min followed by 60 cycles of 94 °C for 15 s, 60 °C for 30 s, and 72 °C for 30 s, after which the samples were heated to 95 °C for 1 min, 55 °C for 1 min, and then held at 4 °C. The assay was performed with a volume of 20 μL in 96-well plates using a MyiQ Real-Time Single Color PCR Detection System (Bio-Rad Laboratories Inc., Hercules, CA, USA). Relative quantification of gene expression was analyzed using the 2-ΔΔ*C*T method [22].

### 2.7. Statistics

Data are expressed as the mean ± SD. All statistical analyses were performed using SPSS 11.5 (SPSS Inc., Chicago, IL, USA). Comparisons between two groups and multiple groups were analyzed using Student’s *t*-test or one-way ANOVA, respectively. *P*-value less than 0.05 was considered statistically significant.

## 3. Results and Discussion

**Figure 1:**
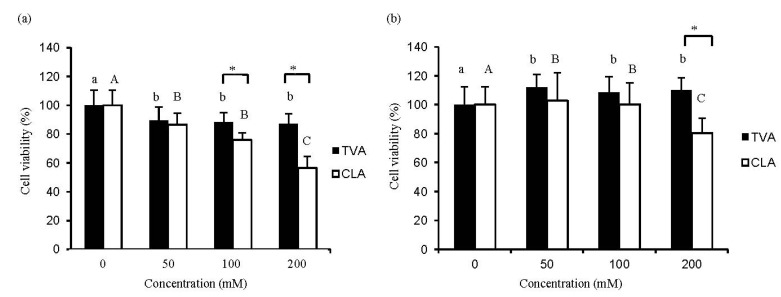
Changes in the number of MCF-7 (**a**) or MCF-10A (**b**) cells after exposure to trans vaccenic acid (TVA) or c9, conjugated linoleic acid (t11-CLA) at the concentration of 0, 50, 100 and 200 mM for 72 h. Cell growth was measured by the uptake of MTT dye and absorbance at 550 nm. Results of the MTT data were analyzed using a one-way ANOVA. SD values are shown as error bars (*n* = 8). Lower cases indicate significantly different (*p* < 0.05) in TVA group; while the upper cases indicate significantly different (*p* < 0.05) in CLA group. The asterisk indicates significantly different (*p* < 0.05) between TVA group and CLA group.

The proliferation of human breast cancer MCF-7 cells and human mammary epithelial MCF-10A cells after exposure to various concentrations of TVA and c9, t11-CLA for 72 h were measured. MTT assay results revealed that the cell viability of MCF-7 cells was inhibited in a dose-dependent manner (*p* < 0.05) when treated with different concentrations of TVA. Growth inhibitory ratios of MCF-7 cells treated with 50, 100, and 200 μM of c9, t11-CLA were 13.9%, 22.7%, and 47.6% (*p* < 0.05), respectively. In addition, when treated with the same concentrations (100 μM and 200 μM), c9, t11-CLA was more effective than those of the TVA (*p* < 0.05) (Figure 1a). The number of MCF-10A cells exposure to c9, t11-CLA at concentrations of 50, 100, and 200 μM was reduced by 2.7%, 0.2%, and 19.7% (*p* < 0.05), respectively. In contrast, the number of MCF-10A cells incubated with TVA at concentrations of 50, 100, and 200 μM was increased approximately 12.0%, 8.0%, and 9.9% (*p* < 0.05), respectively (Figure 1b). One previous study reported that MCF-7 growth was inhibited by incubation with TVA (25 μg/mL) for 96 h. TVA supplementation for 4 days at concentrations of 5, 10, and 15 μg/mL had no effect on cell growth, whereas 20 μg/mL significantly (*p* < 0.05) reduced the growth of MCF-7 cells [9]. In another study, the viability of T47D breast carcinoma cells was significantly decreased by treatment with both TVA and c9, t11-CLA (50–200 μM) [23].

**Figure 2:**
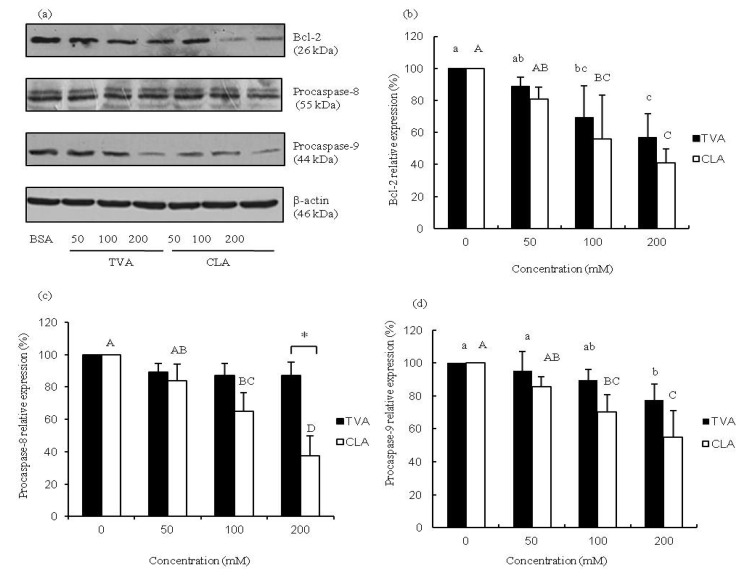
Representative protein expression images (**a**) bar diagram of (**b**) Bcl-2; (**c**) caspase-8; and (**d**) procaspase-9 in MCF-7 cells exposed to TVA or c9, t11-CLA for 72 h. Western blotting data were analyzed using a one-way ANOVA. SD values are shown as error bars (*n* = 3). Lower cases indicate significantly different (*p* < 0.05) in TVA group; while the upper cases indicate significantly different (*p* < 0.05) in CLA group.

The process of apoptosis is controlled by a diverse range of cell signals that may originate from two major cell-intrinsic pathways: one that begins with ligation of cell surface cell receptors and another that involves the release of cytochrome c from the mitochondrial intra-membrane space into the cytoplasm. Previous studies suggested that CLA may regulate the expression of the major oncogenes involved in cell survival and programmed cell death in human cancer cells, and alter the expression of key pro-apoptotic genes such as p53, p21, WAF/CIP1, and Bcl-2 [12,14]. Furthermore, it has been reported that CLA reduces the protein levels of the anti-apoptotic factor Bcl-xl, the translocation of cytochrome c from mitochondria to the cytosol, and the cleavage of procaspase-9 and procaspase-3 [23]. The results of our study indicated that TVA at concentrations of 100 and 200 μM, similar to CLA, induces the down-regulation of Bcl-2 protein expression in MCF-7 cells (Figure 2a,b). c9, t11-CLA (100 and 200 μM) suppressed the expression of both procaspase-8 and -9 proteins whereas reduced protein levels of procaspase-9 were observed in MCF-7 cells exposed to 200 μM TVA (Figure 2c,d). These results were similar to those of some previous reports [14,24]. Our results indicated that CLA may trigger apoptosis via mechanisms involving the entire mitochondrial pathway mentioned above.

**Figure 3:**
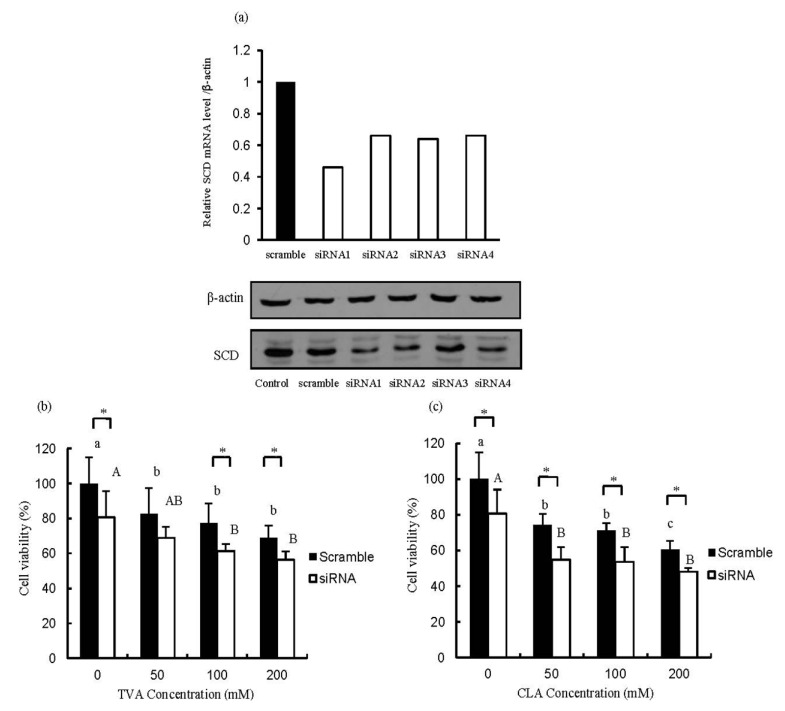
Efficient suppression of stearoyl CoA desaturase (SCD) mRNA expression after siRNA transfection (**a**) and changes in the number of SCD-depleted MCF-7 cells after exposure to (**b**) TVA or (**c**) c9, t11-CLA. MTT data were analyzed using a one-way ANOVA. SD values are shown as error bars (*n* = 8). Values not labeled with the same letter are significantly different (*p* < 0.05). Lower cases indicate significantly different (*p* < 0.05) in scramble group; while the upper cases indicate significantly different (*p* < 0.05) in siRNA group. The asterisk indicates significantly different (*p* < 0.05) between scramble group and siRNA group. CCTACGCCACCAATTTCGT (scrambled), GAGAUAAGUUGGAGACGAU (siRNA1), GAUAUGCUGUGGUGCUUAA (siRNA2), GGGCATAACAGCAGGAGCT (siRNA3), and GGAGATGGAAACTA CAAGA (siRNA4).

As shown in Figure 3a the SCD protein expression was decreased after specific SCD-siRNA transfected compared to that of the scrambled siRNA. TVA significantly inhibited the growth of MCF-7 cells transfected with siRNA1 compared with that transfected with the scrambled siRNA (Figure 3b). On the other hand, c9, t11-CLA did not significantly affect the proliferation of MCF-7 cells transfected with either scrambled siRNA or siRNA1. The effect of SCD knockdown on cell viability was clearly observed in the BSA-treated groups (Figure 3b,c). A previous research in our lab demonstrated that TVA owned suppressive effect on T47D breast carcinoma cells [23]. All these results suggested that TVA may have a direct anti-carcinogenic effect on human mammary adenocarcinoma cells and the clinical application of TVA on cancer prevention can be expected. In this manuscript, the SCD1 was knockdown (>50%) by specific siRNA but not knockout. However, from the data collected in this study, the anti-carcinogenesis effect of TVA could be speculated. In order to check the direct anti-carcinogenesis effect of TVA, four weeks’ administration of 0.5 g/100 g dietary TVA on the physiological condition of mice compared with those of equal c9, t11-CLA are underway in our lab now. In addition, further study utilizing SCD1 knockout mouse have also been designed in our lab. 

## 4. Conclusions

TVA significantly suppressed the proliferation of MCF-7 cells by down-regulating the protein expression of Bcl-2 and procaspase-9. Suppression of cell proliferation by TVA was observed in siRNA1-transfected MCF-7 cells, which suggested that TVA may exert a direct anti-carcinogenic effect on human mammary adenocarcinoma cells. Our findings provided a better understanding of the research on the anti-carcinogenic effects of TVA.
